# Acute hemorrhagic rectal ulcer: Experience in 11 patients at an urban acute care center in the USA: A case series

**DOI:** 10.1097/MD.0000000000019836

**Published:** 2020-05-01

**Authors:** Choichi Sugawa, Ashley Culver, Mark Diebel, Jennifer S. McLeod, Charles E. Lucas

**Affiliations:** Wayne State University School of Medicine, Detroit Receiving Hospital, Michael & Marian Ilitch Department of Surgery, 4201 St. Antoine Avenue, Room 6C, Detroit, Michigan 48201, USA.

**Keywords:** acute hemorrhagic rectal ulcer in USA, critical illness, lowers gastrointestinal bleeding, treatment of AHRU

## Abstract

**Introduction:**

Acute hemorrhagic rectal ulcer (AHRU) is a rare entity which has most frequently been described in Japan and Taiwan literature. This study characterizes 11 AHRUs identified and managed at an urban acute care hospital in the United States of America (USA).

**Methods:**

A total of 2253 inpatients underwent colonoscopy. In 1172 patients (52%), colonoscopy was performed for evaluation of lower gastrointestinal (LGI) bleeding. Eleven (0.9%) of the 1172 patients with LGI bleeding had AHRU.

**Results:**

AHRU is characterized by a sudden onset of painless and massive lower rectal bleeding in elderly, bedridden patients (pts) with major underlying diseases. The endoscopic findings were classified into 4 types. All 11 ulcers were located in the distal rectum within 10 cm of the dentate line. All 11 patients required blood transfusion (mean = 3.7 units; range 2–9 units). Seven patients responded to blood, plasma, and platelet transfusions. The other 4 patients required endoscopic hemostasis.

Three patients died within a month of colonoscopy from comorbidities. None had bleeding as a cause of death. Eight surviving patients did not have recurrent bleeding.

**Conclusion:**

AHRU does exist in the USA and should be considered as an important cause of acute lower GI bleeding in elderly, critically ill, and bedridden patients. AHRU should be recognized and managed correctly.

## 1 Introduction

Acute hemorrhagic rectal ulcer (AHRU) is a rare entity which has most frequently been described in the Eastern world. The term, AHRU, was coined by Soeno and co-authors in 1981 in the Japanese literature.^[1]^ They described this syndrome, in 4 patients with cerebral ischemia, by the acute onset of painless and massive rectal bleeding. This syndrome typically occurs in elderly bedridden patients. Endoscopic examination confirms the AHRU as the source of life-threatening bleeding. Most subsequent reports through 2018 emanated from the eastern world, primarily out of Japan and Taiwan.^[2–11]^ Delaney and Hitch, in 1974 (3 patients),^[12]^ and Duff and Wright, in 1981 (7/9 acute bleeding patients)^[13]^ described in Western world what is now called AHRU although they did not use the term “AHRU”.

A recent experience in 2010 with a case of AHRU in an elderly male inpatient stimulated this review. This elderly patient required multiple transfusions and failed angiographic control of massive bleeding. When this failed, he was successfully controlled by endoscopic guided injection of 20 cc of 1:10,000 normal saline and epinephrine solution. This experience instigated this retrospective review of a prospectively generated registry program to identify the incidence, treatment, morbidity, and mortality of this entity.

## 2 Methods

The medical records of 2253 consecutive inpatients, who underwent colonoscopies on the surgical service in an urban emergency hospital between July 1, 2009 and December 31, 2015, were reviewed. Patients who had colonoscopies as an outpatient were excluded. All rectal ulcers were tabulated and divided according to etiology. Patients with associated colitis, (inflammatory bowel disease, pouchitis, and radiation proctitis) were excluded. Electronic medical records, paper endoscopy reports, and endoscopic images were reviewed in detail. The incidence, underlying disorder, comorbidities, endoscopic findings, ambulatory status, presence or absence of anticoagulant or antiplatelet therapy, treatment, and mortality were tabulated. A retrospective chart review was carried out to gain in-depth information regarding patient information and therapeutic interventions with the approval of the Wayne State University Institutional Review Board, under IRB# 080816MP2E, indicating informed consent for each patient was not needed.

### 2.1 Inclusion and exclusion criteria of AHRU

Those patients who fulfilled all of the following criteria were considered to have AHRU; sudden onset of painless, massive rectal bleeding; (2) serious underlying disorders (e.g., respiratory failure, renal failure, liver failure, diabetes mellitus, cerebrovascular accident (CVA), atherosclerosis); (3) presence of benign ulcerations in the rectum with ongoing bleeding or stigmata of recent bleeding confirmed by colonoscopy; (4) absence of fecal impaction in the rectum; (5) absence of colitis (inflammatory bowel disease, pouchitis, and radiation proctitis); (6) absence of other rectal ulcerative disorders such as solitary rectal ulcer syndrome, stercoral ulcer, ischemic colitis, or mechanical injuries (enema or rectal tube related).^[3,7,8]^

## 3 Results

### 3.1 Incidence of acute hemorrhagic rectal ulcers and causes of rectal ulcers

A total of 2253 inpatients underwent colonoscopy including 90 patients who had flexible sigmoidoscopy. In 1172 patients (52%), colonoscopy was performed for evaluation of lower gastrointestinal (LGI) bleeding. Fifty-four of the 2253 patients (2.4%) had rectal ulcers; 11 (0.5%) of those 2253 patients with ulcers met the criteria of AHRU. Eleven (0.9%) of the 1172 patients with LGI bleeding had AHRU.

The types of rectal ulcers in these 54 patients (Table 1) included stercoral (14), enema or rectal tube induced (13), AHRU (11), solitary (3), human immunodeficiency virus (HIV) (3), rectal injury (gunshot wound) (3), cocaine (2), ischemic (2), pseudomonas (1), nonbleeding acute rectal ulcer (1), and idiopathic (1). The 1 elderly patient (81 years old) with an acute rectal ulcer without hemorrhage was also bedridden and had multiple comorbidities without constipation or pain. This patient was found to be bleeding from a duodenal ulcer as seen by esophagogastroduodenoscopy (EGD). The idiopathic ulcer was a 2 cm × 2 cm deep rectal ulcer about 12 cm above the dentate line (Table 1). This patient with idiopathic ulcer was a healthy, ambulatory, middle-aged woman from Bangladesh, and her ulcer did not fit any criteria described above. Stercoral ulcers were sometimes difficult to differentiate from AHRU by colonoscopic findings only; the history of chronic constipation and very hard stool identified the stercoral ulcers.

**Table 1:**
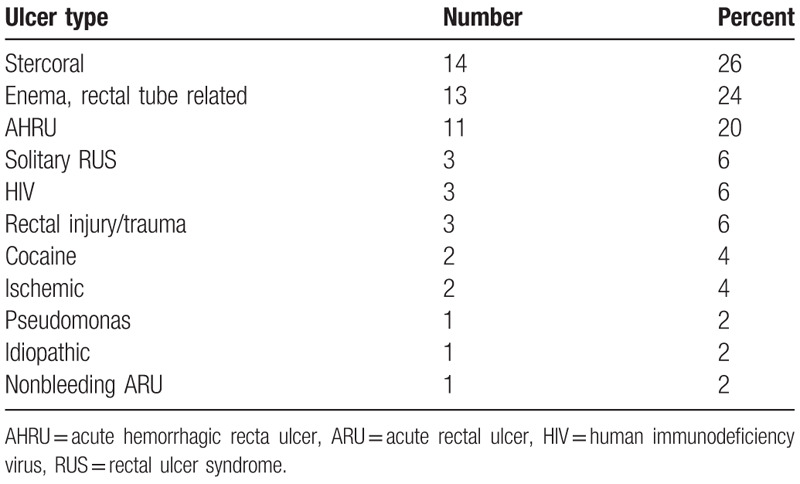
Causes of rectal ulcers 54/2253 inpatients (2.4%).

### 3.2 Clinical features

All 11 AHRU patients, 5 men and 6 women, had painless hematochezia (Table 2). The mean age was 65 years (range 45–88), and all were treated in the intensive care unit (ICU). All 11 patients were bedridden. Ten out of 11 patients were in the ICU when they started having severe painless LGI bleeding. The major underlying diseases included end stage renal disease in 8 patients, hypertension in 7 patients, diabetes mellitus in 4 patients, acute CVA in 3 patients, and liver failure in 3 patients. Five of the patients were being treated with anticoagulants and/or antiplatelet agents.

**Table 2:**
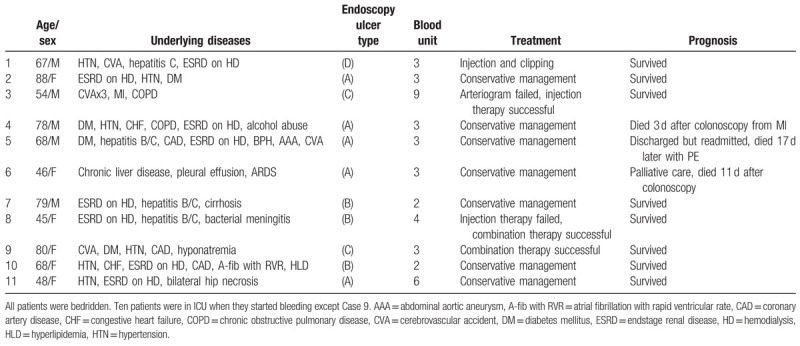
Clinical characteristics of 11 patients with AHRU – chronological order.

### 3.3 Endoscopic findings

**Figure 1:**
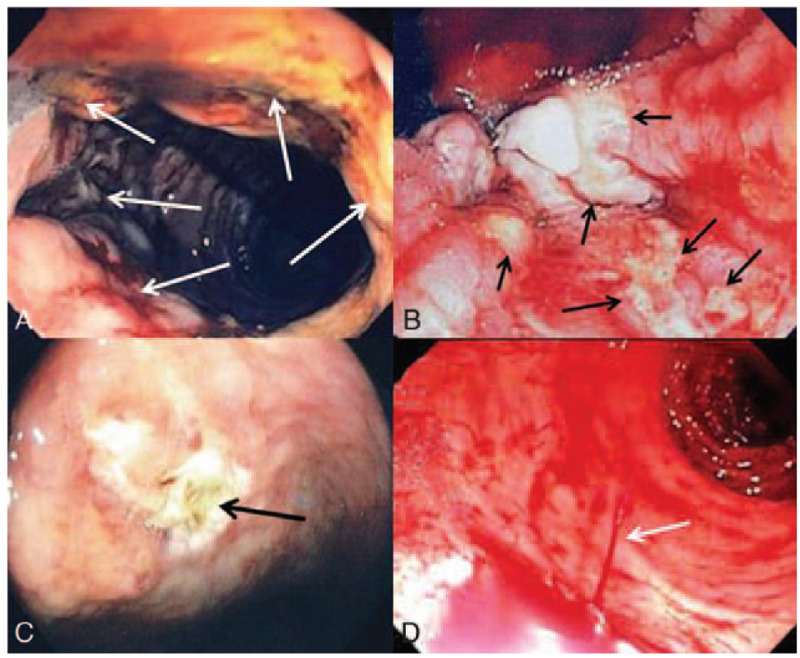
A: This patient has a circumferential ulcer with the extent identified by the arrows; this ulcer extends 8 cm from the anal verge. Note the necrotic edges on the left side as the ulcer with blood and blood clot extends up into the rectum (Case 5) (Type A ulcer). B: This image shows multiple small ulcers identified by the arrows near the dentate line typical of a type B AHRU (retroflexed view) (Case 8). Initial injection therapy failed but combination therapy was successful. C: Endoscopic picture of type C AHRU showing 1 large irregular ulcer 5 cm above the dentate line (Case 9). This shows an ulcer after successful hemostasis by combination therapy. D: This image shows active bleeding (arrow) with a stream of blood coming from a pinpoint area in the mucosa with normal surrounding mucosa just inside of the dentate line. This patient was successfully treated by injection and clipping. This is a classic type D AHRU (Case 1).

The endoscopic findings in these 11 patients were classified by the system described by Motomura.^[8]^ These included 5 patients with long circumferential ulcers (Type A, Fig. 1A); 4 patients with multiple small ulcers near or at the dentate line (Type B, Fig. 1B); 1 patient with round and irregular ulcers (Type C, Fig. 1C); and 1 patient with a Dieulafoy-like lesion (Type D, Fig. 1D) (Table 2). All ulcers were located in the distal rectum within 10 cm of the dentate line.

### 3.4 Treatment and prognosis

All 11 patients required blood transfusion (mean = 3.7 units; range 2–9 units). Seven patients responded to blood, plasma, and platelet transfusions (Table 2). The other 4 patients required endoscopic hemostasis consisting of epinephrine injection alone (2 patients (pts)), epinephrine injection followed by heater probe (1 pt), and epinephrine injection followed by endoclipping (1 pt). Endoscopic hemostasis was initially successful in these 4 patients. Recurrent bleeding in 1 patient after epinephrine injection alone was successfully controlled by a combination of epinephrine injection and heater probe. Three patients died within a month of colonoscopy from comorbidities. None had bleeding as a cause of death. Eight surviving patients did not have recurrent bleeding.

## 4 Discussion

The most common cause of LGI bleeding is diverticular disease, followed by hemorrhoids, carcinoma or polyps, and angiodysplasia.^[14–17]^ The reported incidence of severe lower gastrointestinal bleeding from rectal ulcers is 2 to 21 percent.^[10,11,15,17]^ Bleeding from rectal ulcers has been recognized and distinguished from the other etiologies of LGI bleeding over the past many years, but the term “acute hemorrhagic rectal ulcer” was seldom used in Western Hemisphere publications.^[14–16]^. AHRU has now been accepted as a specific entity and includes the ulcers18 described by Delaney and Hitch in 1974^[12]^ and Hendrickson and coworkers in 2003.^[19]^ Kanwal and coworkers, in 2003, reported on 23 patients from the United States of America (USA) with bleeding rectal ulcers, including 16 patients with major comorbidities.^[20]^ They opined that the etiology was idiopathic in 11 of these 23 patients; in retrospect some of these may have been AHRU. Hendrickson and coworkers,^[19]^ also in 2003, described 4 inpatients with multiple comorbidities who had severe massive rectal bleeding from rectal ulcers in 1 institution during a 6-month period. Those reports from USA have not use the term “AHRU”. AHRU may be underreported due to difficulties in the localization of causes of rectal bleeding.^[5,18,19]^

The critical illnesses of our patients reflected the overall syndrome of AHRU; the common major comorbidities included end stage renal failure, hypertension, severe diabetes mellitus, liver failure, and cardiac failure or arrhythmia, often with ongoing therapy with anticoagulants or antiplatelet agents (Table 2). All 11 patients were bedridden. These clinical features coincide with the many reports in the literature. With the acceptance of AHRU as a distinct entity, more recent reports demonstrate that this is the most common cause of massive rectal bleeding in patients being managed in the intensive care unit.^[9]^ The report herein supports that observation, as 10 of 11 patients developed AHRU during their ICU hospitalization.

Tseng and coworkers divided the endoscopic findings of AHRU into 3 categories, namely round type, geographic type, and circumferential type.^[4]^ Oku and coworkers also had a 3-type classification defined as “nearly” round type, irregular type, and Dieulafoy type.^[7]^ The classification by Motomura and coworkers^[8]^ has 4 categories, including circumferential type, small linear/round type, circumferential plus small ulcer type, and Dieulafoy-like type. Motomura's classification was used in this report with some modification (Fig. 1A–D). Among the patients with AHRU, those with whole circumferential ulcers (Type A) should be considered for especially high risk of delayed ulcer healing and rebleeding.^[10]^

One of the challenges in classifying AHRU was distinguishing this entity from other bleeding rectal lesions. The endoscopic appearance of some stercoral ulcers could be similar to AHRU^[21]^ but the clinical scenario was much different. The stercoral ulcers were associated with severe constipation and the presence of impacted, brick hard stool at the margin of the ulcer.^[22,23]^ Some bleeding from a stercoral ulcer is difficult to control transanally due to the impacted stool and the difficulty exposing transabdominally without resecting that portion of the proximal rectum and sigmoid colon that was solidly impacted with very hard stool.^[23]^ Endoscopic findings of ischemic ulcers could also be difficult to distinguish from AHRU on endoscopic examination.^[24]^ These ischemic ulcers were more likely to be associated with vascular compromise to other parts of the gut and have the classical computed tomography (CT) scan finding of ischemic bowel. The 2 patients, reported herein, with ischemic ulcers of the rectum also had proximal sigmoid and descending colon ulceration plus the typical CT scan findings of ischemia associated with abdominal pain. Endoscopic findings from AHRU could be similar to the Dieulafoy-like lesions which typically would have a protruding vessel, with a very small, less than 3 mm, mucosal defect.^[25,26]^ One of the patients reported herein had a Dieulafoy-like lesion with projectile bleeding from a small ulcer near the dentate line which showed otherwise normal mucosa. Acute rectal ulcer related to an enema or recently placed rectal tube usually has a single, or few longitudinal, fresh laceration plus the history of the recent use of a rectal tube, enema, or other foreign body. An inflated rectal tube may produce a deep ulcer near the dentate line. The solitary rectal ulcer was more likely to be associated with rectal prolapse after straining in patients with pelvic floor incoordination caused by chronic constipation.^[27,28]^ The histologic features of a solitary rectal ulcer (fibromuscular obliteration) are not found in patients with AHRU. The rectal ulcer caused by HIV was suspected by the presence of HIV, the colonoscopic findings, and histopathologic evaluation.^[29]^ There were 2 patients in this series who had ulcers due to cocaine injection. This was associated with ischemic ulceration of many areas of the gut and was suspected by the recent use of cocaine. Cocaine is known to cause intense vasoconstriction with ischemia and localized perforation with or without bleeding.^[30]^

Nakamura reported that the rectal mucosal blood flow was actually reduced in the supine position.^[2]^ This was measured by laser-Doppler technique. They postulated that this reduction of mucosal blood flow is a major etiology of AHRU.^[2]^ Ischemic rectal ulcers were thought to be rare due to the abundant collateral flow around the rectum, but they may develop in patients who have not had vascular surgery or other iatrogenic interventions such as sclerotherapy or hemorrhoidal therapy.^[31]^ Rectal ischemia can occur in conjunction with or without colonic ischemia and represents about 10 to 15 percent of all cases of large bowel ischemia.^[17]^ Ischemic colitis could also present as painless or painful hematochezia with left-sided abdominal discomfort.^[17]^ The painless subtype of ischemia typically results from mucosal hypoxia and is thought to be caused by hypoperfusion of the intramural vessels of the colonic wall.^[17]^ In the study reported herein, hypertension and/or stroke were present in 8 patients, and there were 4 patients with diabetes who might have had compromised flow. The elderly age in most patients suggested that arterial sclerosis may be a potential risk factor. Bleeding tendencies were also found in 8 patients being treated with end stage renal disease. Furthermore, 5 patients were being treated with anticoagulants or antiplatelet agents. The combination of poor rectal perfusion and coagulopathy in the bedridden,^[10]^ arteriosclerotic patient played a role in the onset of massive painless bleeding from AHRU.

Many types of treatment for AHRU have been applied since ulcer types of AHRU are multiple. Transanal gauze tamponade may be initially successful but has a significant recurrence rate.^[4]^ Transanal suture ligation of an actively bleeding vessel, ideally, would be an excellent technique, but access to the bleeding vessel through a limited orifice and actual isolation of the artery may be very frustrating and result in failure to achieve good hemostasis.^[6]^ Clipping of a visible vessel has been reported with success, whereby both hypertonic saline injection and band ligation have been used.^[7,8]^ Application of a heater probe has also been reported with some success^[9]^. Endoscopic hemostasis in this report was achieved in 4 of 11 patients with excellent results. The technique for successful hemostasis in these patients included clipping, epinephrine/saline injection, heater probe, and bipolar electrocoagulation.

The treatment in this series included 7 patients who responded to blood plasma and platelet transfusion without the need for endoscopic hemostasis. The other 4 patients required endoscopic hemostasis, which was successful. Unfortunately, the comorbidities in these patients were major, so 3 patients died within 1 month of presentation. None of the patients died from continuous bleeding. The surviving 8 patients responded to treatment and did not rebleed. This coincides with prior reports showing that the patient outcome is influenced by underlying diseases rather than continued bleeding.^[4,7,9]^

### 4.1 Limitations of the present study

Limitations of the present study include that it was carried out at a single emergency hospital and the number of AHRU was small. Nevertheless, this study shows that AHRU exists in the USA. AHRU should be considered as an important cause of acute LGI bleeding in patients with a severe underlying illness.

## 5 Conclusion

AHRU does exist in the USA and should be considered as an important cause of acute LGI bleeding in elderly, critically ill, and bedridden patients. AHRU should be recognized as an important cause of severe rectal bleeding and should be managed correctly.

## Author contributions

Choichi Sugawa designed, analyzed, wrote, and revised this manuscript; Ashley Culver analyzed the data and helped in the revision of this manuscript; Mark Diebel and Jennifer S. McLeod collected the data for this manuscript; Charles E. Lucas is responsible for the critical editing in the revision of this manuscript.
